# Light propagation in inhomogeneous media, coupled quantum harmonic oscillators and phase transitions

**DOI:** 10.1038/s41598-019-53024-5

**Published:** 2019-11-14

**Authors:** Alejandro R. Urzúa, Irán Ramos-Prieto, Francisco Soto-Eguibar, Víctor Arrizón, Héctor M. Moya-Cessa

**Affiliations:** 1 Instituto Nacional de Astrofísica, Óptica y Electrónica, Calle Luis Enrique Erro No. 1, Santa María Tonantzintla, Puebla 72840 Mexico; 2 Instituto de Ciencias Físicas, Universidad Nacional Autónoma de México, Apartado Postal 48-3, 62251 Cuernavaca, Morelos Mexico

**Keywords:** Optics and photonics, Optical physics

## Abstract

This contribution has two main purposes. First, using classical optics we show how to model two coupled quantum harmonic oscillators and two interacting quantized fields. Second, we present classical analogs of coupled harmonic oscillators that correspond to anisotropic quadratic graded indexed media in a rotated reference frame, and we use operator techniques, common to quantum mechanics, to solve the propagation of light through a particular type of graded indexed medium. Additionally, we show that the system presents phase transitions.

## Introduction

The existence of analogies between quantum and classical mechanics has been applied for many years, particularly in the generation of mathematical tools to provide solutions of optical problems and vice versa^1–11^. The reason is that the optical paraxial wave equation is mathematically equivalent to the stationary Schrödinger equation and, on the other hand, Helmholtz equation has a treatment similar to that of time-independent Schrödinger equation. Exploring analogies between different physical problems is useful, as it allows exporting insights from one context to the other.

Classical optics systems have been used to model quantum optical phenomena^6^. There has been also a proposal by Man’ko *et al*.^12^ to realize quantum computation by using quantum-like systems. Coherent random walks have been shown to occur in free propagation provided the initial wave function is tailored properly^13^. The modelling of time-dependent harmonic oscillators has been achieved in a graded indexed (GRIN) media^14^ allowing to show the splitting of modes in second order solutions of the Helmholtz equation^15^. The propagation of classical fields in a graded indexed medium with linear dependence has shown to produce and control Airy modes^16^.

On the other hand, much attention has been recently devoted to the study of phase transitions^17,18^ in the Rabi Model^19,20^. By means of the Holstein-Primakoff transformation^21^, it may be argued that the interaction of two quantized fields is similar to the Rabi interaction, when we approximate the spin-flip operators as creation and annihilation operators and the energy Pauli matrix as a harmonic oscillator, and thus the two interacting fields can show phase transitions.

Being aware of all of these correspondences, in this contribution we apply methods commonly used in quantum optics to solve a problem of classical optics. Although, we could use variable separation to solve the propagation of light in GRIN media, we believe it is of interest to use operator techniques, commonly used in quantum mechanics, as there may be cases in which the refraction index not only depends on *x* and *y* but also on the propagation distance *z* (see for instance Urzúa *et al*.^22^), and then the separation of variables is not possible.

In particular, we show in the next section that classical light propagation in an inhomogeneous medium models a coupled quantum harmonic oscillators or the interaction of two quantized fields. In the same section, we also show the conditions for a phase transition of the coupled system, in the sense that the set of parameters involved lead one of the oscillators go through harmonic to free particle and finally to a repulsive oscillator. In the Section *Invariant modes*, we show that it is possible to obtain invariant modes in the classical propagation of light in the specific inhomogeneous medium considered. In the Section titled *Helmholtz equation*, we briefly discuss the possibility of splitting a beam by taking into account the fact that, to second order in the Helmholtz equation, a kind of quantum Kerr medium is modelled. Finally, the last section is left for conclusions.

## Paraxial Wave Equation for Inhomogeneous Media

There exist classical optical systems related to waveguiding of optical modes that may be described by the paraxial wave equation

1 $$2{\rm{i}}{k}_{0}\frac{\partial E}{\partial z}={\nabla }_{\perp }^{2}E+{k}^{2}(x,y)E,$$

where *λ* and *k*_0_ = 2*πn*_0_/*λ* are the wavelength and the wavenumber of the propagating mode, respectively, and *n*_0_ is the homogeneous refraction index. The function *k*^2^(*x*, *y*) describes the inhomogeneity of the medium responsible for the waveguiding of the optical field *E*. The inhomogeneity may be physical, in the sense that it is produced in the medium when fabricated; an example of such a medium is a graded index fiber. In particular, we will consider^23^

2 $${k}^{2}(x,y)={k}_{0}^{2}-({k}_{x}{x}^{2}+{k}_{y}{y}^{2})+2gxy,$$

being *k*_*x*_, *k*_*y*_ and *g* parameters that characterize the inhomogeneous medium. In ref.^23^, it was proposed a way, that could be easy to realize experimentally, of generating the inhomogeneity function *k*^2^(*x*, *y*) by the co-propagation of two modes in a Kerr nonlinear medium.

Equation (1), which is analogous in structure to the Schrödinger equation^24^, together with the inhomogeneity (2), has been solved for the special case of $g\ll {k}_{x},{k}_{y}$ using the so-called rotating wave approximation^23^; here, we solve it for any set of parameters. We may rewrite Eq. (1) as

3 $${\rm{i}}\frac{\partial E}{\partial z}=\hat{H}E$$

with

4 $$\hat{H}=-\frac{1}{2{k}_{0}}({\hat{p}}_{x}^{2}+{\hat{p}}_{y}^{2})+\frac{1}{{k}_{0}}[\frac{{k}_{0}^{2}}{2}-\frac{1}{2}({k}_{x}{x}^{2}+{k}_{y}{y}^{2})+gxy],$$

where the momentum operators have the usual definition on configuration space, ${\hat{p}}_{x}=-\,{\rm{i}}\frac{\partial }{\partial x}$ and ${\hat{p}}_{y}=-\,{\rm{i}}\frac{\partial }{\partial y}$. By considering the unitary operator

5 $${\hat{R}}_{\theta }=\exp [{\rm{i}}\theta (x{\hat{p}}_{y}-y{\hat{p}}_{x})]$$

we make the transformation $E={\hat{R}}_{\theta }^{\dagger } {\mathcal E}$, such that we obtain a Schrödinger-like equation for ${\mathcal E}$ as

6 $${\rm{i}}\frac{\partial  {\mathcal E} }{\partial z}=\frac{1}{2{k}_{0}}({k}_{0}^{2}-{\hat{p}}_{x}^{2}-{\tilde{k}}_{x}{x}^{2}-{\hat{p}}_{y}^{2}-{\tilde{k}}_{y}{y}^{2}+\tilde{g}xy) {\mathcal E} ,$$

where

7a $${\tilde{k}}_{x}=\frac{{k}_{x}+{k}_{y}}{2}+\frac{{k}_{x}-{k}_{y}}{2}\,\cos (2\theta )-g\,\sin (2\theta ),$$

7b $${\tilde{k}}_{y}=\frac{{k}_{x}+{k}_{y}}{2}-\frac{{k}_{x}-{k}_{y}}{2}\,\cos (2\theta )+g\,\sin (2\theta ),$$

7c $$\tilde{g}=({k}_{x}-{k}_{y})\sin (2\theta )+2g\,\cos (2\theta ),$$

were obtained from the set of transformations

8a $${\hat{R}}_{\theta }x{\hat{R}}_{\theta }^{\dagger }=x\,\cos \,\theta -y\,\sin \,\theta ,$$

8b $${\hat{R}}_{\theta }y{\hat{R}}_{\theta }^{\dagger }=y\,\cos \,\theta +x\,\sin \,\theta ,$$

8c $${\hat{R}}_{\theta }{\hat{p}}_{x}{\hat{R}}_{\theta }^{\dagger }={\hat{p}}_{x}\,\cos \,\theta -{\hat{p}}_{y}\,\sin \,\theta ,$$

8d $${\hat{R}}_{\theta }{\hat{p}}_{y}{\hat{R}}_{\theta }^{\dagger }={\hat{p}}_{y}\,\cos \,\theta +{\hat{p}}_{x}\,\sin \,\theta .$$

From Eq. (7c), we choose $\theta =\frac{1}{2}\arctan (\frac{2g}{{k}_{y}-{k}_{x}})$ and we have

9 $$\cos (2\theta )=\frac{{k}_{y}-{k}_{x}}{\sqrt{{({k}_{y}-{k}_{x})}^{2}+4{g}^{2}}},\,\,\sin (2\theta )=\frac{2g}{\sqrt{{({k}_{y}-{k}_{x})}^{2}+4{g}^{2}}},$$

and then Equations (7) take the form

10a $${\tilde{k}}_{x}=\frac{{k}_{x}+{k}_{y}}{2}-\frac{1}{2}\sqrt{{({k}_{x}-{k}_{y})}^{2}+4{g}^{2}},$$

10b $${\tilde{k}}_{y}=\frac{{k}_{x}+{k}_{y}}{2}+\frac{1}{2}\sqrt{{({k}_{x}-{k}_{y})}^{2}+4{g}^{2}},$$

10c $$\tilde{g}=0,$$

so that (6) is transformed into the equation of two uncoupled quantum harmonic oscillators

11 $${\rm{i}}\frac{\partial  {\mathcal E} }{\partial z}=[\frac{{k}_{0}}{2}-\frac{1}{2{k}_{0}}({\hat{p}}_{x}^{2}+{\tilde{k}}_{x}{x}^{2})-\frac{1}{2{k}_{0}}({\hat{p}}_{y}^{2}+{\tilde{k}}_{y}{y}^{2})] {\mathcal E} ,$$

for which we know the formal solution.

**Figure 1:**
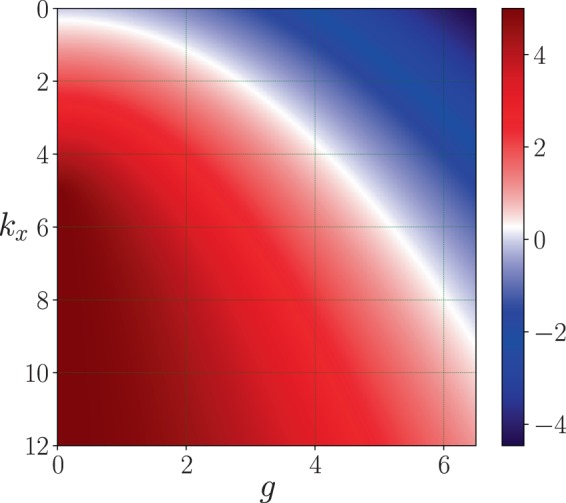
Plot surface of  ${\tilde{k}}_{x}$ as a function of *k*_*x*_ and *g*, with *k*_*y*_ = 5. We see that there is a phase transition from positive to negative values of  ${\tilde{k}}_{x}$ when *g* goes to bigger values respect to the former.

We may see from Eq. (10a), that for some values of the parameters *k*_*x*_, *k*_*y*_ and *g* of the inhomogeneity of the medium, the effective frequency of the oscillator, ${\tilde{k}}_{x}$, goes from positive to negative (see Fig. 1), therefore undergoing a phase transition^17,18^. It basically shows that the oscillator in the variable *x* goes from a harmonic oscillator to a repulsive harmonic oscillator, passing through a free particle^25^. In other words, if we define a critical value for *g*,

12 $${g}_{c}=\sqrt{{k}_{x}{k}_{y}},$$

we have the following three cases:

13 $$\begin{array}{l}g < {g}_{c}\,\,{\rm{Harmonico}}\,{\rm{scillator}},\\ g={g}_{c}\,\,{\rm{Free}}\,{\rm{particle}},\\ g > {g}_{c}\,\,{\rm{Repulsive}}\,{\rm{oscillator}}.\end{array}$$

If in Eq. (6), we write *x* and *y* in terms of annihilation and creation operators, we see that the so-called counter rotating terms have not been neglected (i.e., the rotating wave approximation was not performed). The presence of those terms is responsible for the phase transition in the propagation of light in the GRIN (Gradient-index) medium we are considering^23^.

**Figure 2:**
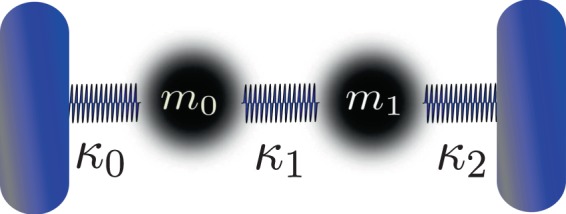
Two masses coupled by springs, which is described by the Hamiltonian (15). This system exhibits the same features found in the Hamiltonian (4) for the light propagation in an inhomogeneous medium.

A mechanical system equivalent to the light propagation analyzed above is given by two masses connected between them by springs and connected to physical or mathematical constrains (like a pair of walls) also by springs, as it is depicted in Fig. (2). The equations that rule this system are^26^

14a $${\kappa }_{1}({q}_{1}-{q}_{0})-{\kappa }_{0}{q}_{0}={m}_{0}{\ddot{q}}_{0},$$

14b $$-{\kappa }_{2}{q}_{1}-{\kappa }_{1}({q}_{1}-{q}_{0})={m}_{1}{\ddot{q}}_{1},$$

where *q*_0_, *q*_1_ are the positions of mass *m*_0_ and mass *m*_1_, respectively, and *κ*_0_, *κ*_1_, *κ*_2_ are the three spring constants. Introducing the Hamiltonian

15 $$H=\frac{{p}_{0}^{2}}{2{m}_{0}}+\frac{{p}_{1}^{2}}{2{m}_{1}}+\frac{1}{2}{\kappa }_{0}{q}_{0}^{2}+\frac{1}{2}{\kappa }_{2}{q}_{1}^{2}+\frac{1}{2}{\kappa }_{1}{({q}_{1}-{q}_{0})}^{2},$$

in the Hamilton equations, performing some derivatives and some algebra, we get the system (14). So, (15) is indeed a Hamiltonian of the system illustrated in Fig. (2).

We can make now the connection between our model of light propagation with this mechanical system. If in Eq. (3) we make *z* → −*t* and

$${k}_{0}={m}_{0}={m}_{1}=m,\,\,{k}_{x}=m\frac{{\kappa }_{0}+{\kappa }_{1}}{2},\,\,{k}_{y}=m\frac{{\kappa }_{1}+{\kappa }_{2}}{2},\,\,g=m{\kappa }_{1},$$

the two Hamiltonians, (4) and (15), are identified. We can go also in the other sense, from the mechanical system to the light propagation model, making *t* → −*z* and

$${m}_{0}={m}_{1}=m={k}_{0},\,\,{\kappa }_{0}=\frac{g-2{k}_{x}}{m},\,\,{\kappa }_{1}=\frac{g}{m},\,\,{\kappa }_{2}=\frac{g-2{k}_{y}}{m}.$$

## Invariant Modes

It is easy to show that there exist invariant modes (without dependence on the propagation) for the inhomogeneity we are studying, provided that the parameter ${\tilde{k}}_{x}$ is positive, so that we keep ourselves in the regime of an harmonic oscillator. Consider a field at *z* = 0 given by

16 $$E(x,y,z=0)={\hat{R}}_{\theta }^{\dagger }{\psi }_{{n}_{x}}(x){\psi }_{{n}_{y}}(y),$$

where *ψ*_*nx*_(*x*) and *ψ*_*ny*_(*y*) are Hermite-Gauss functions; i.e., eigenfunctions of the harmonic oscillators given in (11). This allows us to write the initial transformed field (16) as ${\mathcal E} (x,y,z\,=\,\mathrm{0)}\,=\,{\psi }_{{n}_{x}}(x){\psi }_{{n}_{y}}(y)$ and therefore write the solution of Eq. (11) as

17 $$ {\mathcal E} (x,y,z)=\exp (-{\rm{i}}\frac{{k}_{0}z}{2})\,\exp \,[{\rm{i}}\frac{z}{{k}_{0}}({\omega }_{x}+{\omega }_{y})]\,\exp \,[{\rm{i}}\frac{z}{{k}_{0}}({n}_{x}{\omega }_{x}+{n}_{y}{\omega }_{y})]\,{\psi }_{{n}_{x}}(x){\psi }_{{n}_{y}}(y),$$

that produces the propagated field

18 $$E(x,y,z)=\exp (-{\rm{i}}\frac{{k}_{0}z}{2})\,\exp \,[{\rm{i}}\frac{z}{{k}_{0}}({\omega }_{x}+{\omega }_{y})]\,\exp \,[{\rm{i}}\frac{z}{{k}_{0}}({n}_{x}{\omega }_{x}+{n}_{y}{\omega }_{y})]\,{\hat{R}}_{\theta }^{\dagger }{\psi }_{{n}_{x}}(x){\psi }_{{n}_{y}}(y),$$

that is clearly an invariant mode with associated phases depending on the angular quantities ${\omega }_{x}=\sqrt{{\tilde{k}}_{x}}$ and ${\omega }_{y}=\sqrt{{\tilde{k}}_{y}}$, and in the independent energy levels *n*_*x*_ and *n*_*y*_. The way the operator ${\hat{R}}_{\theta }$ acts on the Hermite-Gauss functions on (18) is detailed in the Appendices.

**Figure 3:**
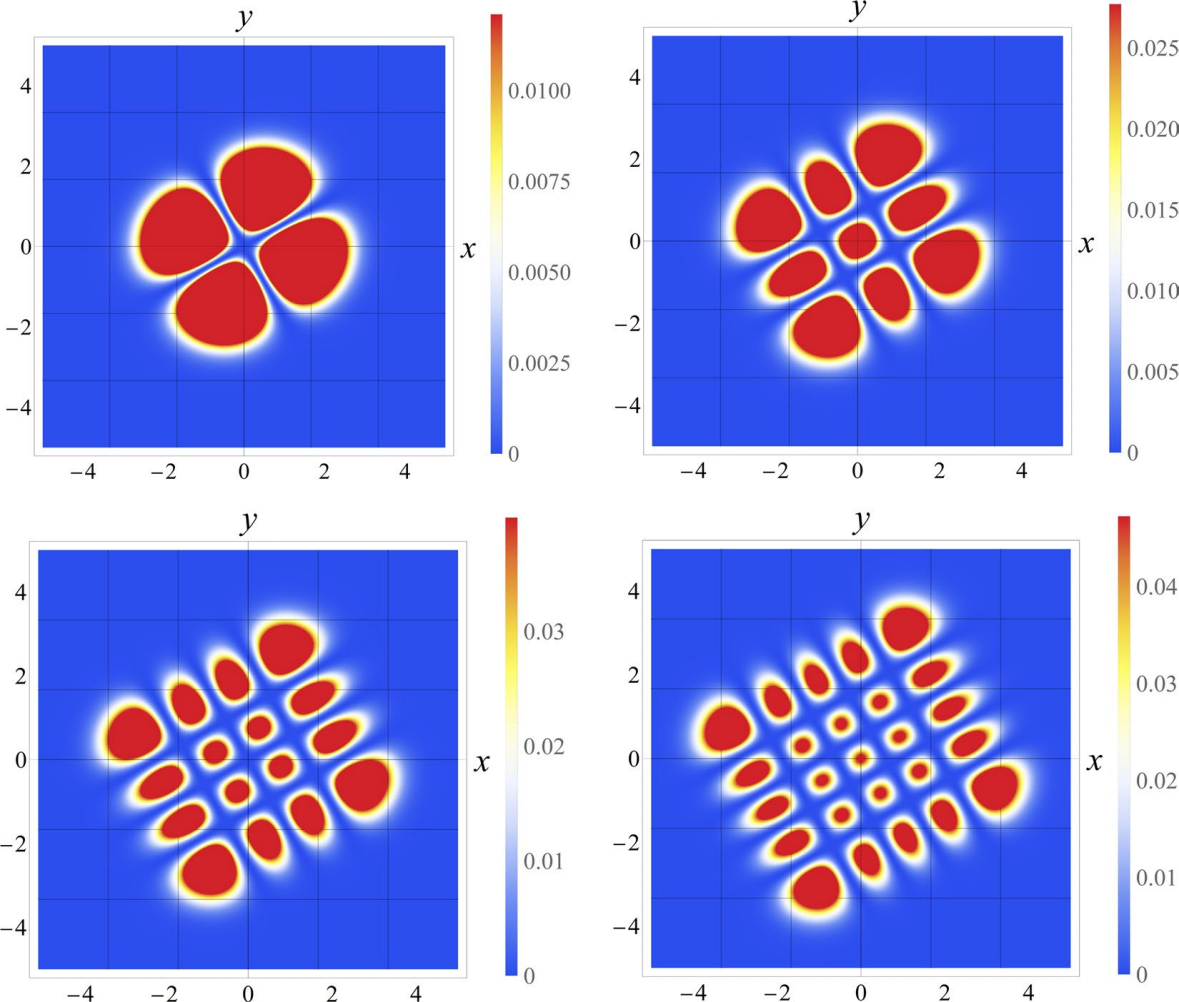
Plot of the square modulus of (18) for different values of *n*_*x*_ and *n*_*y*_. Upper left: *n*_*x*_ = *n*_*y*_ = 1, upper right: *n*_*x*_ = *n*_*y*_ = 2, lower left: *n*_*x*_ = *n*_*y*_ = 3, lower right: *n*_*x*_ = *n*_*y*_ = 4. For all the cases *k*_*x*_ = 1.2, *k*_*y*_ = 1.5 and *g* = 0.25. The propagation is invariant along the propagation axis, i.e., it does not depend on the propagation distance *z*, despite the selection of the energy levels.

Some examples of the behavior of the propagated field *E*(*x*, *y*, *z*) in (18) are shown in Fig. 3.

It is convenient to stress that although the propagation is invariant, there are some values of the coefficients {*k*_*x*_, *k*_*y*_, *g*} for which there is a phase transition and therefore the parameter ${\tilde{k}}_{x}$ goes from positive (an usual harmonic oscillator) to negative (a repulsive harmonic oscillator). In the negative region, see Fig. 1, the Hermite-Gauss functions are not anymore eigenfunctions of the repulsive harmonic oscillator, therefore an invariant mode is not produced. As it was pointed out by Yuce^27^ and Muñoz^28^, the eigenfunctions of the repulsive harmonic oscillator are a combination of the eigenfunctions of the free particle due to the existence of a equivalence between the repulsive potential and the free evolution through a proper canonical transform. Since these eigenfunctions are a combination of plane waves and waves confined in a box or propagating to a potential barrier, the non invariance argument above when *g* > *g*_*c*_ follows.

## Helmholtz Equation

We consider now the complete Helmholtz equation

19 $$\frac{{\partial }^{2}E}{\partial {z}^{2}}=-[\frac{{\partial }^{2}}{\partial {x}^{2}}+\frac{{\partial }^{2}}{\partial {y}^{2}}+{k}^{2}(x,y)]E.$$

For the case we considered above, in the section titled *Paraxial wave equation for inhomogeneous media*, and doing the same transformation (5), we arrive to

20 $$E(x,y,z)=\exp [-{\rm{i}}z\sqrt{\frac{{k}_{0}}{2}-\frac{{\omega }_{x}}{{k}_{0}}({n}_{x}+\frac{1}{2})-\frac{{\omega }_{y}}{{k}_{0}}({n}_{y}+\frac{1}{2})}]E(x,y,0),$$

that may be developed to second order in *κ*^2^ = *k*_0_^2^ − *k*_*x*_ − *k*_*y*_ to obtain the approximation

21 $$E(x,y,z)=\exp [-{\rm{i}}z\frac{\kappa }{\sqrt{2{k}_{0}}}(1-\frac{{\rm{\Omega }}}{2{\kappa }^{2}}-\frac{{{\rm{\Omega }}}^{2}}{8{\kappa }^{4}})]E(x,y,0),$$

where

22 $${\rm{\Omega }}={k}_{x}+{k}_{y}-{\omega }_{x}(2{n}_{x}+1)-{\omega }_{y}(2{n}_{y}+1).$$

This equation allows the splitting of fields^29^ as the square terms resemble nonlinear quantum optical interactions, namely a quantum Kerr medium^30^.

## Conclusions

We have shown that we can model the interaction of two masses through springs, i.e. two coupled quantum harmonic oscillators and the interaction of two quantized fields in a graded index media. The solutions we have given to second order approximation of the Helmholtz equation show how a beam splitter may be achieved in such media. We have also studied that phase transitions occur in the propagation of light in this medium, by showing that one of the harmonic oscillators is inverted for certain values of the parameters involved.

### Factorization of the operator *R̂*_*θ*_

In this appendix the unitary operator

23 $${\hat{R}}_{\theta }=\exp [{\rm{i}}\theta (\hat{x}{\hat{p}}_{y}-\hat{y}{\hat{p}}_{x})]$$

is factorized.

We define the operators

24 $${\hat{K}}_{+}=\hat{x}{\hat{p}}_{y},$$

25 $${\hat{K}}_{-}=\hat{y}{\hat{p}}_{x},$$

26 $${\hat{K}}_{0}={\rm{i}}\frac{1}{2}(\hat{x}{\hat{p}}_{x}-\hat{y}{\hat{p}}_{y}).$$

The exponential of the operator ${\hat{K}}_{0}$ is nothing but a product of squeeze operators^31–35^. The above operators have the commutators

27 $$[{\hat{K}}_{+},{\hat{K}}_{-}]=-\,2{\hat{K}}_{0},$$

28 $$[{\hat{K}}_{0},{\hat{K}}_{+}]={\hat{K}}_{+},$$

29 $$[{\hat{K}}_{0},{\hat{K}}_{-}]=-\,{\hat{K}}_{-},$$

and thus generate a su(1, 1) algebra. We find the factorization

30 $${\hat{R}}_{\theta }=\exp [{\rm{i}}\theta ({\hat{K}}_{+}-{\hat{K}}_{-})]=\exp ({\rm{i}}{f}_{1}{\hat{K}}_{+})\exp ({\rm{i}}{f}_{2}{\hat{K}}_{0})\exp ({\rm{i}}{f}_{3}{\hat{K}}_{-})$$

where *f*_1_, *f*_2_, *f*_3_ are given by

31 $${f}_{1}(\theta )=\,\tan \,\theta ,\,\,{f}_{2}(\theta )=2{\rm{i}}\,\mathrm{ln}(\cos \,\theta ),\,\,{f}_{3}(\theta )=-\,\tan \,\theta $$

or, writing it explicitly in terms of the original operators,

32 $$\begin{array}{c}{\hat{R}}_{\theta }=\exp [{\rm{i}}\theta (\hat{x}{\hat{p}}_{y}-\hat{y}{\hat{p}}_{x})]=\exp [{\rm{i}}\,\tan (\theta )\hat{x}{\hat{p}}_{y}]\\ \,\,\,\exp \{-{\rm{i}}\,\mathrm{ln}[\cos (\theta )](\hat{x}{\hat{p}}_{x}-\hat{y}{\hat{p}}_{y})\}\exp [-{\rm{i}}\,\tan (\theta )\hat{y}{\hat{p}}_{x}].\end{array}$$

### Action of the operator *R̂*_*θ*_ over a function *F*(*x*, *y*)

It is not difficult to show that

33 $${\hat{R}}_{\theta }F(x,y)=F[\cos (\theta )x-\,\sin (\theta )y,\,\sin (\theta )x+\,\cos (\theta )y],$$

where ${\hat{R}}_{\theta }$ is given in (32) and *F*(*x*, *y*) is an arbitrary, but well behaved, function of *x* and *y*.

To study the action of the ${\hat{R}}_{\theta }$ operator over an arbitrary function *F*(*x*, *y*), we make

34 $${\hat{R}}_{\theta }=\exp [{\rm{i}}\theta (\hat{x}{\hat{p}}_{y}-\hat{y}{\hat{p}}_{x})]={\hat{T}}_{y}{\hat{S}}_{xy}{\hat{T}}_{x},$$

where

35 $${\hat{T}}_{y}=\exp [{\rm{i}}\,\tan (\theta )\hat{x}{\hat{p}}_{y}],$$

36 $${\hat{S}}_{xy}=\exp \{-{\rm{i}}\,\mathrm{ln}[\cos (\theta )](\hat{x}{\hat{p}}_{x}-\hat{y}{\hat{p}}_{y})\}$$

37 $${\hat{T}}_{x}=\exp [-{\rm{i}}\,\tan (\theta )\hat{y}{\hat{p}}_{x}].$$

Note that the operator ${\hat{S}}_{{xy}}$ is a product of squeeze operators^31–35^ in *x* and *y*. We can prove that

38 $${\hat{T}}_{x}F(x)=F[x-\,\tan (\theta )y],\,\,{\hat{T}}_{y}G(y)=G[y+\,\tan (\theta )x],$$

and the action of the squeeze operators on the variables *x* and *y* are

39 $$\exp ({\rm{i}}r\hat{x}{\hat{p}}_{x})x=\exp (r)x,\,\,\exp ({\rm{i}}r\hat{y}{\hat{p}}_{y})y=\exp (r)y.$$
